# Impact of Chronic Mild Stress on Neurotrophic and Hypothalamic-Pituitary-Adrenal Factors in the Brain of Rats Submitted to Sepsis

**DOI:** 10.1007/s12640-026-00801-6

**Published:** 2026-05-23

**Authors:** Jefté Peper-Nascimento, Taise Possamai-Della, Thais Gois-Carvalho, Jorge M. Aguiar-Geraldo, Bruna Pescador, João Quevedo, Felipe Dal-Pizzol, Samira S. Valvassori

**Affiliations:** 1 Translational Psychiatry Laboratory, Graduate Program in Health Sciences, University of Southern Santa Catarina (UNESC), Criciúma, SC Brazil; 2 Center for Interventional Psychiatry, Faillace Department of Psychiatry and Behavioral Sciences, McGovern Medical School, The University of Texas Health Science Center at Houston (UTHealth Houston), Houston, TX USA; 3 Laboratory of Experimental Pathophysiology, Graduate Program in Health Sciences, University of Southern Santa Catarina (UNESC), Criciúma, SC Brazil

**Keywords:** Sepsis, Depression, Neurotrophic Factors, Neurotrophins, Chronic Mild Stress

## Abstract

The present study aimed to investigate the effects of chronic mild stress (CMS) on neurotrophic factors in the brains of rats subjected to the cecal ligation and puncture (CLP) model of sepsis. After 30 days of the CLP procedure, the animals were subjected to CMS. Twenty-four hours after the last stressor, the animals were euthanized, their blood was collected, and their brains were dissected in the frontal cortex and hippocampus. Then, the levels of brain-derived neurotrophic factor, neurotrophin-3, neurotrophin-4, nerve growth factor, and glial cell line-derived neurotrophic factor were evaluated in the brain structures. The levels of adrenocorticotropic hormone and corticosterone were evaluated in the serum of the rats. The four experimental groups of the present study were: (1) Sham + Non-stressed; (2) Sepsis + Non-stressed; (3) Sham + CMS; (4) Sepsis + CMS. CMS increased adrenocorticotropic hormone and corticosterone levels, whereas sepsis alone did not significantly alter these hormones. Regarding neurotrophic factors, CMS reduced the levels of BDNF, NT-3, NT-4, and NGF in both the hippocampus and frontal cortex, regardless of prior sepsis exposure. Sepsis alone was also associated with reduced levels of these neurotrophins, although the magnitude of the effect varied depending on the specific neurotrophin and brain region, with greater reductions observed for BDNF and NGF. No interaction between sepsis and CMS was detected for these neurotrophins, suggesting independent effects. Our findings suggest that sepsis and CMS independently affect most neurotrophin levels, with no clear evidence that prior sepsis potentiates the effects of subsequent CMS. GDNF represents an exception, pointing to potential region-specific mechanisms that warrant further investigation.

**Supplementary Information:**

The online version contains supplementary material available at 10.1007/s12640-026-00801-6.

## Introduction

Sepsis is a life-threatening organ dysfunction resulting from a dysregulated host response to infection (Singer et al. 2016). It represents one of the leading causes of mortality in critically ill patients, with an estimated annual incidence of approximately 49 million cases and 11 million deaths worldwide, accounting for nearly 20% of all global deaths (Singer et al. 2016; Sakr et al. 2018; Rudd et al. 2020). Furthermore, survivors of sepsis often experience long-term physical, psychological, and cognitive impairments (Iwashyna et al. 2010).

Sepsis triggers a massive systemic immune response, characterized by the release of cytokines such as interleukin (IL)-1, IL-6, and tumor necrosis factor-alpha (TNF-α), along with the activation of inflammasomes (Danielski et al. 2020; Addissouky et al. 2023). The central nervous system is among the most vulnerable systems affected by sepsis progression, resulting in alterations to blood-brain barrier permeability, oxidative balance, cellular processes, mitochondrial function, and mechanisms of neuroplasticity (Caraballo and Jaimes 2019; Pan et al. 2022; Yang et al. 2025).

The neuroinflammation and alterations in neuroplasticity induced by sepsis appear to co-occur with long-term psychiatric disorders, including depressive symptoms (Li et al. 2024). These mechanisms represent important factors linked to the pathophysiology of depression (Beurel et al. 2020). Preclinical sepsis models, such as lipopolysaccharide (LPS) administration, can induce depressive-like behavior in certain mouse strains and are used to investigate the inflammatory mechanisms associated with the disorder (Yin et al. 2023). Furthermore, the cecal ligation and puncture (CLP) model, considered the gold standard for inducing sepsis-like alterations in rodents, has been shown to elicit depressive-like behavior in survivor rats (Barichello et al. 2007) and to alter neurotrophic factor levels in the brain (Aguiar-Geraldo et al. 2025). These alterations affect key neurotrophic factors such as brain-derived neurotrophic factor (BDNF), nerve growth factor (NGF), and glial cell line-derived neurotrophic factor (GDNF) (Aguiar-Geraldo et al. 2025). The relationship between long-term depressive-like symptoms and sepsis-induced neurotrophic changes remains incompletely understood; however, the activation of stress signaling pathways may link both conditions.

The chronic mild stress (CMS) protocol is a widely used animal model for inducing depressive-like behavior. The biochemical alterations associated with CMS-induced behavioral symptoms include changes in neurotrophins levels, inflammatory parameters, and hypothalamic-pituitary-adrenal (HPA) axis activity (Willner 2017; Pan et al. 2022). Although previous research has demonstrated that CMS prior to CLP can attenuate neuroinflammation and depressive-like symptoms (Steckert et al. 2017), the effects of CMS on animals with pre-existing vulnerability due to sepsis remain poorly characterized. Elucidating this interaction may uncover key mechanisms contributing to post-sepsis psychiatric outcomes. Therefore, this study aimed to investigate the impact of CMS on the brains of rats subjected to the CLP model of sepsis.

## Materials and Methods

### Animals

This study used 30-day-old male Wistar rats from the University of Southern Santa Catarina colony. The animals were allocated in five animals per cage (41 × 35 × 16 cm), under controlled temperature (22 ± 1 °C), relative humidity (45–55%), and day/light cycle (12:12 h, light on at 06:00 h). The rats had free access to food (standard diet for laboratory animals - NUVILAB CR-1^®^, Brazil) and water. All experiments were conducted in accordance with the National Institutes of Health (NIH) Guide for the Care and Use of Laboratory Animals and the guidelines of the National Council for the Control of Animal Experimentation (CONCEA). The study was approved by the local Ethics Committee of the University of Southern Santa Catarina (protocol no. 051-2014-02).

### CLP Model

The rats were anesthetized intraperitoneally (i.p.) with ketamine (80 mg/kg) and xylazine (10 mg/kg). Then, a 3-cm midline laparotomy was performed to allow exposure of the cecum to the adjoining intestine. The cecum was tight with a 3.0-silk suture, ligated at its base, below the ileocecal valve, and perforated once with a 14-gauge needle. The cecum was then squeezed gently to extrude a small amount of feces from the perforation site and then returned to the peritoneal cavity. With 4.0-silk sutures, the laparotomy was closed. After this process, all animals returned to their cages with free food and water. The animals of the sham-operated group were submitted to all surgical procedures, but the cecum was neither ligated nor perforated. After surgery, the sepsis group received “basic support” (50 mL/kg saline immediately and 12 h after CLP plus 30 mg/kg ceftriaxone and 25 mg/kg clindamycin every 6 h for 3 days. Following the CLP procedure, rats were housed in small groups of 2 in a temperature-controlled environment (approx. 25 °C) to prevent hypothermia, a common complication of sepsis. Soft bedding was provided, and food and hydration (including subcutaneous saline bolus for fluid resuscitation) were placed in easily accessible locations on the cage floor to facilitate recovery for animals with limited mobility. The sham-operated group received only 50 mL/kg saline immediately, and 12 h after surgery, and the volume of saline corresponded to antibiotic administration (Hubbard et al. 2005). Rats subjected to CLP had a mortality rate of 30%, similar to that reported by Garcia et al. (2023).

### CMS Model

**Fig. 1:**
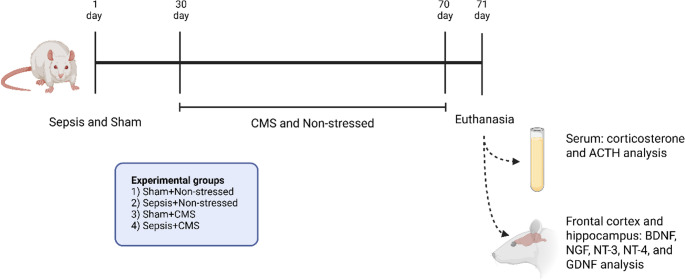
Schematic representation of the experimental design of the present study. Rats were subjected to the CLP model to induce sepsis. Following CLP, rats received basic support (saline) and antibiotic therapy (ceftriaxone + clindamycin) for 3 days. The SHAM group underwent the same surgical procedure but did not receive antibiotics post-CLP. Thirty days after surgery, rats were divided into two groups: a non-stressed group and a group subjected to a 40-day CMS protocol, which involved daily exposure to unpredictable stressors. These included: (I) 24 h food deprivation; (II) 24 h water deprivation; (III) 1–3 h of restraint; (IV) 1.5–2 h of restraint at 4 °C; (V) 120–210 min of flashing light; and (VI) isolation for 2–3 days. Euthanasia was performed 24 h after the final stressor. Thereafter, serum samples were collected for corticosterone and adrenocorticotropic hormone (ACTH) analysis, and frontal cortex and hippocampus samples were dissected for analysis of neurotrophic factors (brain-derived neurotrophic factor (BDNF), neurotrophin-3 (NT-3), neurotrophin-4 (NT-4), nerve growth factor (NGF), and glial cell line-derived neurotrophic factor (GDNF) levels

Thirty days after the CLP procedure, the rats were submitted to the CMS model (Fig. 1**)**. The CMS protocol was adapted from the procedure described by Gamaro et al. (2003). Animals were divided into two groups: non-stressed and CMS (stressed) rats. Animals were submitted to stressors for 40 days. The following stressors were used: (I) 24 h of food deprivation; (II) 24 h of water deprivation; (III) 1–3 h of restraint as described later; (IV) 1.5–2 h of restraint at 4 °C; (V) flashing light for 120–210 min and, (VI) isolation (2–3 days). Stressor stimuli were applied at different times every day to minimize the prediction. Restraint was performed by placing the animal in a 25 × 7 cm plastic tube and securing it with plaster tape on the outside, thus not allowing the rats to move. There was a 1 cm hole at the end of the tube for breathing. Exposure to flashing light was achieved by placing the animal in a 60 × 60 × 25 cm plywood box divided into 16 cells of 15 × 15 × 25 cm with a frontal glass wall. A 40-watt lamp flashing at a frequency of 60 flashes/min. was used. Before and after the exposure to flashing light protocol, the animals were kept in the room with the light off for acclimatization. The isolation procedure was accomplished by housing the animals in individual opaque cages, preventing visual and physical contact with their conspecifics for 2 days. When the animals were exposed to a cold environment, after the protocol, they were warmed using a heater for approximately 30 min (or until they showed no symptoms of hypothermia). A detailed schedule of stressor agents can be found in Online Resource 1.

### Experimental Groups and Samples

The rats were randomly distributed into four groups (*n* = 6 animals per group): (1) Sham + Non-stressed; (2) Sepsis + Non-stressed; (3) Sham + CMS; (4) Sepsis + CMS. Rats were submitted to euthanasia 24 h after the last stressor stimulus. From the cut made by decapitation, peripheral blood samples were collected in microtubes for the subsequent analysis of HPA axis parameters. The serum was obtained by centrifugation at 3000 g for 5 min and then frozen at − 70 °C until the experiment. The brains of rats were dissected in the frontal cortex and hippocampus, rapidly frozen, and stored at − 80 °C until assayed. For biochemical analysis, the left and right hemispheres of the dissected brain regions were pooled. The samples were homogenized in KCl KH2PO4 (12 mM KCl, 0.038 mM KH2PO4, pH = 7.4) to evaluate BDNF, NGF, GDNF, neurotrophin-3 (NT-3), and neurotrophin-4 (NT-4) levels.

### Levels of Corticosterone and Adrenocorticotropic Hormone (ACTH)

Corticosterone levels were determined using enzyme immunoassay kits (from Siemens Healthineers). Serum ACTH concentrations were determined in animals using commercially available kits (Siemens Healthineers). The analysis was performed by a commercial laboratory (Biolabor Laboratório de Análises Clínicas, Criciúma, Brazil). The measurements were evaluated in Siemens IMMULITE immunoassay system. This system uses solid-phase chemiluminescence chemistry for quantification of hormone concentrations. The total volume of the sample injected was 100uL (in duplicate) and the Siemens IMMULITE immunoassay system was calibrated with commercially available controls.

### Protein Determination

All biochemical measures were normalized to the protein content with bovine albumin as a standard (Lowry et al. 1951).

### Measurement of Neurotrophic Factors

For the analysis of neurotrophic factors, brain tissues were homogenized in phosphate-buffered solution (PBS) with 1 mM phenylmethylsulfonyl fluoride (PMSF) and 1 mM ethylene glycolbis (2-aminoethyl ether)-N, N,N’N’-tetraacetic acid (EGTA). The homogenates were centrifuged at 10,000 g for 20 min, and the supernatants were collected to quantify the neurotrophic factor levels. BDNF, NGF, NT-3, NT-4, and GDNF levels in the frontal cortex and hippocampus were evaluated by sandwich enzyme-linked immunosorbent assay using commercial kits according to the manufacturer’s instructions (NGF [Chemicon Millipore (USA) cat. CYT304 sensitivity: 10–15 pg/mL; BDNF [Chemicon Millipore (USA) cat. CYT306 sensitivity: 7.8 pg/mL; GDNF [Biosensis (USA), cat. BEK-2230 sensitivity: 7.8 pg/mL]; NT-3 [Biosensis (USA), cat. BEK-2221 sensitivity: 5 pg/mL]; NT-4 [Biosensis (USA), cat. BEK-2218 sensitivity: 25 pg/mL])

All the samples were homogenized in a Tris/HCl buffer solution pH 7 and then centrifuge at 14,000xg for 30 min. The supernatants were used for the measurement of BDNF, NGF, NT-3, and NT-4. For GNDF levels the supernatants were acidified with 1 N HCl solution until a pH of around 3. After a 15 min incubation, the samples were neutralized. For all the tests a 100uL aliquot was used (in duplicates). All the analyses were measured in a Molecular Devices SpectraMax 384 Plus.

### Statistical Analysis

All the data were tested for normality using the Shapiro-Wilk test. The difference between groups was analyzed using two-way ANOVA followed by Bonferroni’s test. Data are presented in the bar graphs representing the mean ± standard deviation. The results were considered significant when *p* ≤ 0.05. Statistical analysis and graphical designs were performed using GraphPad Prism version 10.2.3 for Windows (GraphPad Software, Boston, Massachusetts, USA; www.graphpad.com).

## Results

**Fig. 2:**
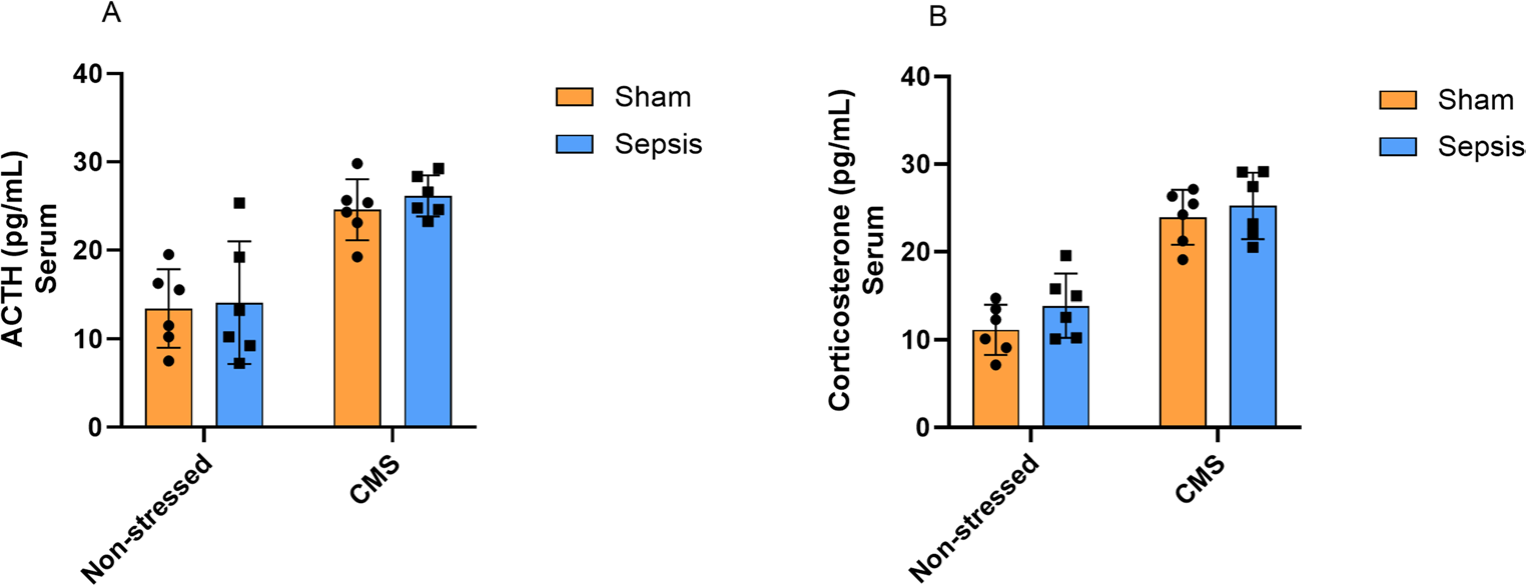
Levels of adrenocorticotropic hormone (ACTH) and corticosterone in the serum of Wistar rats submitted to an animal model of sepsis and the chronic unpredictable stress (CMS) animal model of depression. Data are presented as means ± standard deviation and were analyzed by two-way ANOVA. 2.**A **and 2.**B **(Non-stressed + Sham (*n* = 6), Non-stressed + Sepsis (*n* = 6), CMS + Sham (*n* = 6), and CMS + Sepsis (*n* = 6)

Figure 2 shows the effects of CMS on ACTH and corticosterone levels in rats subjected to the sepsis animal model. For ACTH levels (Fig. 2.A), two-way ANOVA indicated a significant main effect of CMS [F (1, 20) = 38.04, *p* < 0.0001], accounting for 65.09% of the total variance in the data. In contrast, no significant effects were found for sepsis [F (1, 20) = 0.3492, *p* = 0.5612] or for the interaction between CMS and sepsis [F (1, 20) = 0.05613, *p* = 0.8151].

Regarding corticosterone levels (Fig. 2.B), two-way ANOVA similarly showed a significant main effect for the CMS [F (1, 20) = 76.59, *p* < 0.0001], accounting for 77.37% of the total variance observed. In contrast, no significant main effect of sepsis on corticosterone levels was observed [F (1, 20) = 2.141, *p* = 0.1589] nor for the interaction between CMS and sepsis [F (1, 20) = 0.2669, *p* = 0.6111].

**Fig. 3:**
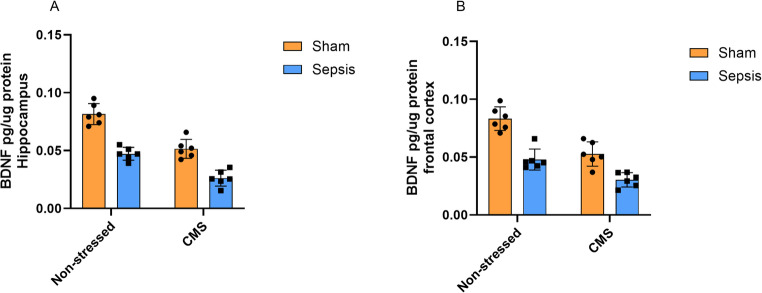
Levels of brain-derived neurotrophic factor (BDNF) in the frontal cortex and hippocampus of Wistar rats submitted to an animal model of sepsis and the chronic unpredictable stress (CMS) animal model of depression. Data are presented as means ± standard deviation and were analyzed by two-way ANOVA. 3.**A** and 3.**B** (Non-stressed + Sham (*n* = 6), Non-stressed + Sepsis (*n* = 6), CMS + Sham (*n* = 6), and CMS + Sepsis (*n* = 6))

Figure 3 shows the levels of BDNF in the hippocampus (3.A) and frontal cortex (3.B) of rats submitted to the CLP model of sepsis and the CMS protocol. In the hippocampus (3.A), a two-way ANOVA revealed significant main effects for both CMS [F(1, 20) = 68.85, *p* < 0.0001], explaining 37.21% of the total variance, and sepsis [F(1, 20) = 94.05, *p* < 0.0001], accounting for 50.83% of the total variance. No significant interaction was observed between CMS and sepsis [F (1, 20) = 2.12, *p* = 0.1608].

Regarding BDNF levels in the frontal cortex (3.B), a two-way ANOVA similarly revealed significant main effects of CMS [F(1, 20) = 41.52, *p* < 0.0001] and sepsis [F(1, 20) = 59.68, *p* < 0.0001], which accounted for 33.41% and 48.03% of the total variance, respectively. The interaction between CMS and sepsis did not reach statistical significance [F (1, 20) = 3.06, *p* = 0.0956].

**Fig. 4:**
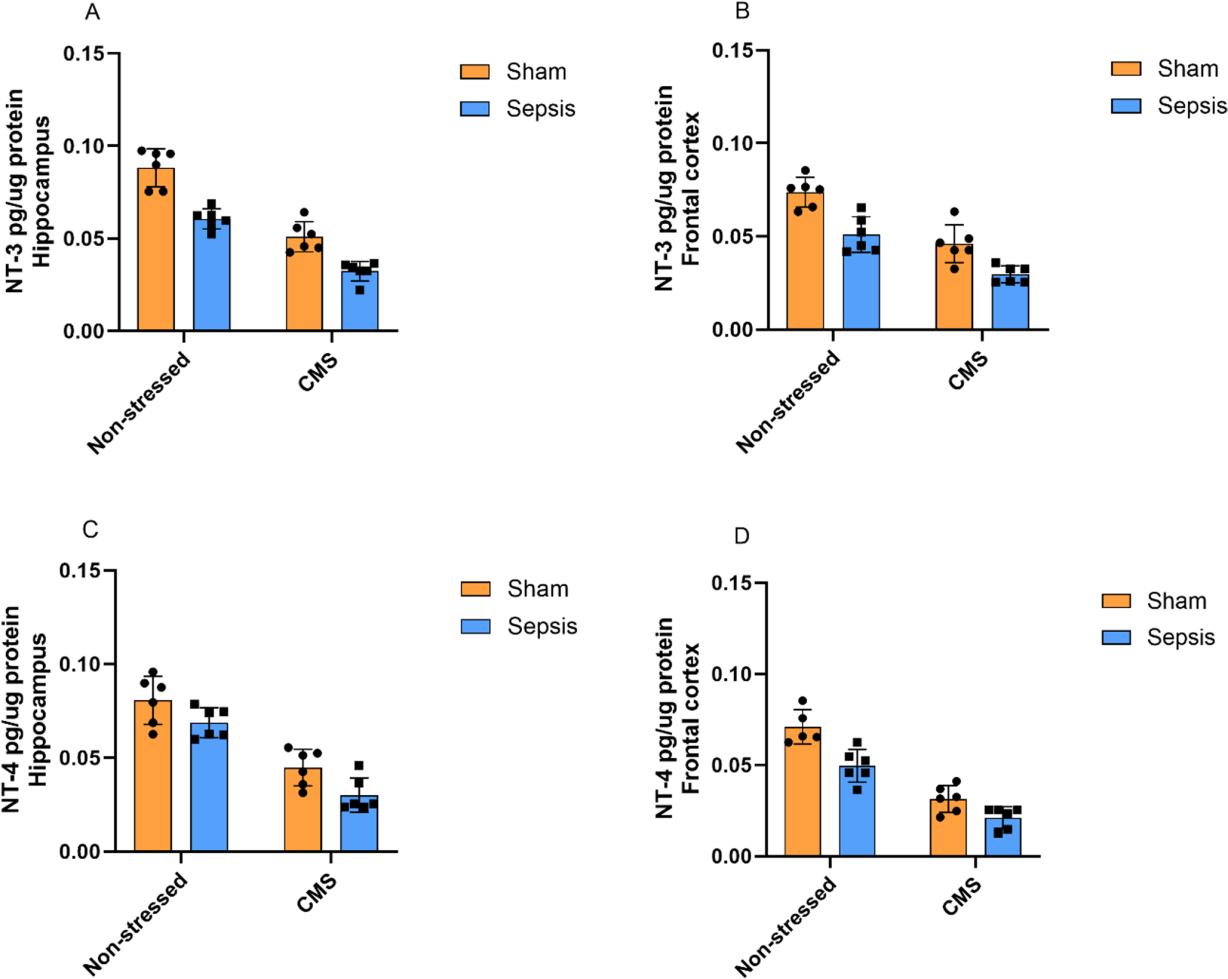
Levels of neurotrophin-3 (NT-3) and neurotrophin-4 (NT-4) in the frontal cortex and hippocampus of Wistar rats submitted to an animal model of sepsis and the chronic unpredictable stress (CMS) animal model of depression. Data are presented as means ± standard deviation and were analyzed by two-way ANOVA. 4.**C **(Non-stressed + Sham (*n* = 6), Non-stressed+ Sepsis (*n* = 6), CMS + Sham (*n* = 6), and CMS + Sepsis (*n* = 6)) and 4.**D **(Non-stressed + Sham (*n* = 5), Non-stressed+ Sepsis (*n* = 6), CMS + Sham (*n* = 6), and CMS + Sepsis (*n* = 6))

Figure 4 shows the levels of NT-3 (4.A and 4.B) and NT-4 (4.C and 4.D). Regarding NT-3 levels in the hippocampus (4.A), a two-way ANOVA revealed significant main effects of CMS [F(1,20) = 112.20, *p* < 0.0001] and sepsis [F(1,20) = 55.92, *p* < 0.0001], which accounted for 58.97% and 29.40% of the total variance, respectively. No significant CMS × sepsis interaction was observed [F(1,20) = 2.11, *p* = 0.1616]. Similar patterns were observed in the frontal cortex (4.B). A two-way ANOVA indicated significant main effects of both CMS [F(1,20) = 51.56, *p* < 0.0001] and sepsis [F(1,20) = 32.81, *p* < 0.0001], which accounted for 49.00% and 31.18% of the total variance, respectively. The CMS × sepsis interaction was not significant [F(1,20) = 0.85, *p* = 0.3671].

Regarding NT-4 levels in the hippocampus (4.C), a two-way ANOVA demonstrated significant main effects of both CMS [F(1,20) = 81.04, *p* < 0.0001] and sepsis [F(1,20) = 10.45, *p* = 0.0037], which accounted for 72.62% and 9.37% of the total variance, respectively. No significant CMS × sepsis interaction was observed [F(1,20) = 0.1024, *p* = 0.7523]. In the frontal cortex (4.D), a two-way ANOVA showed significant main effects for CMS [F(1,19) = 103.80, *p* < 0.0001], explaining 73.20% of the total variance, and sepsis [F(1,19) = 22.36, *p* = 0.0001], responsible for 15.76% of the total variance. No significant interaction between groups was observed [F(1,19) = 2.78, *p* = 0.1116].

**Fig. 5:**
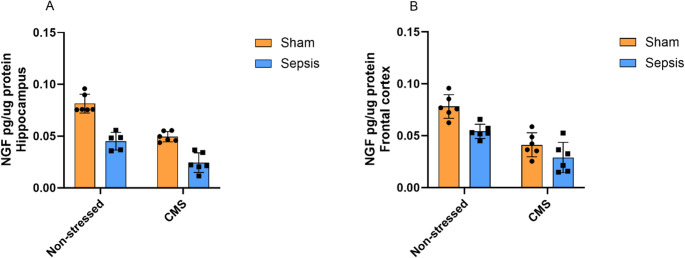
Levels of nerve growth factor (NGF) in the frontal cortex and hippocampus of Wistar rats submitted to an animal model of sepsis and the chronic unpredictable stress (CMS) animal model of depression. Data are presented as means ± standard deviation and were analyzed by two-way ANOVA. 5.**A **(Non-stressed + Sham (*n* = 6), Non-stressed+ Sepsis (*n* = 5), CMS + Sham (*n* = 6), and CMS + Sepsis (*n* = 6)) and 5.**B **(Non-stressed + Sham (*n* = 6), Non-stressed+ Sepsis (*n* = 6), CMS + Sham (*n* = 6), and CMS + Sepsis (*n* = 6))

The levels of NGF are represented in Fig. 5. In the hippocampus (5.A), a two-way ANOVA showed significant main effects for both CMS [F(1,19) = 59.36, *p* < 0.0001], explaining 35.20% of the total variance, and sepsis [F(1,19) = 80.81, *p* < 0.0001], responsible for 47.91% of the total variance. No significant interaction between sepsis and CMS was observed on NGF levels in the hippocampus [F(1,19) = 2.74, *p* = 0.1141]. In the frontal cortex (5.B), a two-way ANOVA demonstrated significant main effects for CMS [F(1,20) = 44.49, *p* < 0.0001], explaining 54.81% of the total variance, and sepsis [F(1,20) = 15.11, *p* = 0.0009], responsible for 18.61% of the total variance. No significant interaction between sepsis and CMS was observed on NGF levels in the frontal cortex [F(1,20) = 1.58, *p* = 0.2231]. Similarly, these findings demonstrate that CMS and sepsis independently decreased NGF levels in the frontal cortex.

**Fig. 6:**
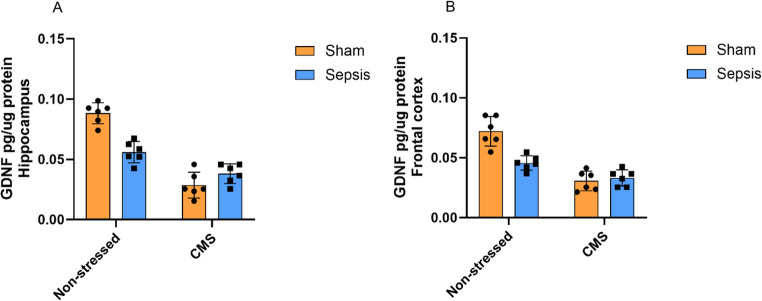
Levels of glial cell line-derived neurotrophic factor (GDNF) in the frontal cortex and hippocampus of Wistar rats submitted to an animal model of sepsis and the chronic unpredictable stress (CMS) animal model of depression. Data are presented as means ± standard deviation and were analyzed by two-way ANOVA followed by Bonferroni’s post-hoc test. 5.**A **(Non-stressed + Sham (*n* = 6), Non-stressed + Sepsis (*n* = 5), CMS + Sham (*n* = 6), and CMS + Sepsis (*n* = 6)) and 5.**B **(Non-stressed + Sham (*n* = 6), Non-stressed + Sepsis (*n* = 6), CMS + Sham (*n* = 6), and CMS + Sepsis (*n* = 6))

Regarding GDNF levels (Fig. 6), a two-way ANOVA revealed a significant interaction between sepsis and CMS on GDNF levels in the hippocampus (6.A) [F(1,20) = 31.41, *p* < 0.0001]. The interaction accounted for 18.59% of the total variance, indicating that the effect of sepsis depended on the presence or absence of subsequent CMS. Pairwise comparisons showed that the Sepsis + CMS group exhibited significantly lower GDNF levels than the Sepsis + Non-stressed group (*p* = 0.017).

In the frontal cortex (6.B), a two-way ANOVA also showed a significant interaction between sepsis and CMS [F(1,20) = 16.76, *p* = 0.0006]. The interaction accounted for 15.74% of the total variance, and post hoc comparisons suggest that the effect of sepsis depended on the presence or absence of CMS. The Sepsis + CMS group exhibited decreased GDNF levels in the frontal cortex compared only with the Non-stressed + Sham group (*p* < 0.001), but had no additional effect in CMS-exposed animals (*p* = 0,1268).

## Discussion

This study investigated the impact of CMS on the HPA axis and neurotrophic factors in the brains of rats previously subjected to sepsis. We first observed that sepsis did not alter ACTH or corticosterone levels when assessed at later time points. In contrast, sepsis reduced the levels of BDNF, NT3, NGF, and GDNF in the frontal cortex and hippocampus, as well as NT4 in the frontal cortex, with effect magnitudes generally smaller than those of stress (except for BDNF and NGF in the hippocampus). The subsequent CMS, in turn, increased ACTH and corticosterone levels both in septic animals and in those exposed only to stress. We also observed that CMS was associated with the greatest reduction in all neurotrophins examined (BDNF, NT-3, NT-4, and NGF in the hippocampus and frontal cortex) and accounted for a substantial proportion of the observed variance. Except for GDNF—which showed a significant sepsis × stress interaction—no significant interactions were found, suggesting that sepsis and CMS exert largely independent effects on neurotrophin regulation. This distinct profile points to a potential involvement of GDNF in mechanisms integrating prior sepsis with later chronic stress.

Homeostatic activation of the HPA axis is essential for survival during sepsis (Goodwin et al. 2013; Annane 2016) as endogenous glucocorticoids counteract key pathological features of the condition, including excessive inflammation, vascular dysfunction, and hypoglycemia (Vandewalle and Libert 2020). In the present study, when assessed in the late phase, sepsis did not induce alterations in ACTH or corticosterone levels in the rat serum. Consistent with our findings, a preclinical study demonstrated that CLP reduced ACTH and corticosterone levels within the first 24 h of sepsis, followed by a gradual recovery after 48 h (Flierl et al. 2011). These results indicate that, when evaluated at later time points, the impact of sepsis on the HPA axis tends to stabilize (Comim et al. 2014; Pereira de Souza Goldim et al. 2020). Thus, HPA axis dysregulation does not appear to persist after disease remission, with ACTH and corticosterone secretion returning to physiological levels when required (dos Santos-Junior et al. 2022).

Neurotrophins such as BDNF, NGF, NT3, and NT4 are essential for neuronal survival and synaptic plasticity, primarily acting through the activation of Trk tyrosine kinase receptors (Bothwell 2014). Increasing evidence indicates that sepsis can induce cognitive dysfunction, neuronal apoptosis, and neuroinflammation, ultimately leading to the development of sepsis-associated encephalopathy (Comim et al. 2014; Tauber et al. 2021; Gao et al. 2022; Fu et al. 2024). Our results show that sepsis was associated with reduced levels of BDNF, NT3, NGF, GDNF, and NT4 in the rat brain. These findings suggest that alterations in neurotrophic factors may persist into the late phase. Moreover, this persistent reduction is associated with the pathophysiology of cognitive impairment both during and after the clinical remission of sepsis, which may contribute to mechanisms underlying long-term neurological deficits (Grünewald et al. 2024; Guo et al. 2025). These alterations have been associated with neuroinflammatory processes triggered by systemic inflammatory responses, in which microglial activation and subsequent cytokine signaling promote neuronal dysfunction and impair hippocampal long-term potentiation (Hoshino 2025). In this context, therapeutic strategies aimed at restoring neurotrophic signaling—such as enriched environment, pharmacological TrkB activation, or interventions targeting neuroinflammation—have emerged as promising approaches to attenuate sepsis-associated cognitive deficits (Zeng et al. 2017; Gao et al. 2022; Grünewald et al. 2024; Xu et al. 2024).

While HPA axis activation constitutes a beneficial physiological response to acute stress and sepsis, its dysregulation is an underlying mechanism of the deleterious effects induced by CMS (Garcia et al. 2009; Nandam et al. 2020; Thakare et al. 2021). In the present study, ACTH and corticosterone levels were affected exclusively by CMS, with no significant effect of sepsis, suggesting that, in this design, prior sepsis did not markedly alter HPA axis reactivity under these conditions. Similarly, numerous preclinical studies have reported increased corticosterone and ACTH levels following exposure to CMS paradigms (Chen et al. 2016; Eliwa et al. 2021; Kim et al. 2023). The production and circulation of glucocorticoids are considered important contributors to depression-like behaviors in response to CMS (Chen et al. 2016).

Prolonged exposure to glucocorticoids and dysfunction of the HPA axis feedback mechanism can lead to neurobiological alterations associated with CMS. These include the modulation of microglial activity, reduced phosphorylation of CREB, and decreased levels of BDNF, among other neurotrophins (Walker et al. 2013; Filho et al. 2015; Willner 2017). In the present study, CMS had a predominant effect on most of the neurotrophins evaluated, particularly NT-4 and cortical NGF. Neurotrophins beyond BDNF, including NT-4, are increasingly recognized as important mediators in the stress response and remain comparatively understudied (Kozlov et al. 2020). The effects of antidepressant treatment on these non-BDNF neurotrophins may be drug-specific; for instance, sertraline has been shown to significantly increase NT-4 and NGF levels in patients with depression (Mishra et al., 2019). In addition, BDNF, its receptor TrkB, and the ERK pathway are thought to play a central role in the stress response (First et al. 2013). Antidepressant drugs have been reported to restore BDNF levels in the frontal cortex (First et al. 2013; Rantamäki 2019). Alterations in neurotrophin signaling and astrocytic function have been linked to anxious and depressive manifestations, potentially related to decreased glucocorticoid receptor expression and increased levels of FKBP5 (Chen et al. 2016). These findings are consistent with the idea that dysfunctions in neurotrophic pathways and glucocorticoid signaling are implicated in the pathophysiology of depression induced by CMS.

Here, the significant interaction observed exclusively for GDNF in both brain regions suggests that this neurotrophin may play a distinct role in integrating the effects of a prior septic insult and a subsequent chronic stressor. In the hippocampus, there was a significant reduction in GDNF in the Sepsis + CMS group when compared to the Sepsis + non-stressed group. Unlike the other neurotrophins evaluated, GDNF belongs to a different superfamily of growth factors and activates intracellular signaling cascades through a specific receptor system involving the GFRα1 coreceptor and the RET tyrosine kinase receptor (Porcari et al. 2025; Saarma 2000). While most neurotrophins signal preferentially through Trk receptors, activation of the RET/GFRα1 pathway by GDNF has been associated with the modulation of diverse physiological processes, including hippocampal synaptic plasticity, glial-mediated neuroinflammatory responses, and distinct neuronal survival mechanisms (Porcari et al. 2025; Khan et al. 2025). This pathway specificity may position GDNF as a potential sensor and modulator in contexts involving multiple challenges to the central nervous system. Importantly, this finding suggests that the impact of chronic stress on central GDNF levels may be modulated by a prior history of sepsis, a pattern of cross-modulation not observed for the other neurotrophic pathways evaluated. Collectively, these results highlight the GDNF/GFRα1/RET system as a potential molecular target for understanding long-term neuroplastic adaptations in sepsis survivors exposed to subsequent stressors.

This study has several important limitations that should be considered when interpreting the findings. First, the exclusive use of male rats prevents the assessment of potential sex differences in the response to stress and sepsis. Second, the analysis focused solely on protein levels of neurotrophic factors, leaving gene expression profiles and the underlying molecular mechanisms unexplored. Another limitation is the absence of pharmacological or behavioral interventions to counteract the deleterious effects of CMS following sepsis, which represents a critical area for future research. Finally, the lack of behavioral analysis restricts the ability to link the observed molecular alterations to depressive-like phenotypes. Incorporating such behavioral assessments in future studies will be essential to enhance the understanding of sepsis-associated depressive-like behavior.

In conclusion, the results indicate that ACTH and corticosterone levels were influenced solely by CMS, with no detectable effect of prior sepsis. In contrast, the majority of neurotrophic markers (BDNF, NT-3, NT-4, and NGF), in both the hippocampus and the frontal cortex, were independently affected by sepsis and CMS. Overall, a greater effect size was associated with stress for most of these variables, except for BDNF and NGF (hippocampus), on which sepsis exerted a more pronounced influence. GDNF was the only neurotrophin to exhibit significant interaction between sepsis and CMS, suggesting the possible involvement of distinct signaling pathways, which may represent a candidate target for therapeutic investigation in clinical conditions where sepsis and chronic stress occur sequentially. Collectively, these findings point to potential region-specific mechanisms involving GDNF that warrant further investigation. 

## Supplementary Information

Below is the link to the electronic supplementary material.

Supplementary Material 1 (DOCX 1.58 MB)

## Data Availability

The datasets generated during and/or analyzed during the current study are not publicly available due to ethical and institutional restrictions imposed by the research ethics committee but are available from the corresponding author on reasonable request.
