# Supplementary material for: Impact of Chronic Mild Stress on Neurotrophic and Hypothalamic-Pituitary-Adrenal Factors in the Brain of Rats Submitted to Sepsis
Source: Neurotox Res. 2026 May 23;44(3):26. doi: 10.1007/s12640-026-00801-6 (PMC13198510; doi:10.1007/s12640-026-00801-6)
Supplement: Supplementary file 1 — Supplementary Material 1 (DOCX 1.58 MB) [file 12640_2026_801_MOESM1_ESM.docx]

Neurotoxicity Research

**Impact of Chronic Mild Stress on Neurotrophic and Hypothalamic-Pituitary-Adrenal Factors in the Brain of Rats Submitted to Sepsis**

Jefté Peper-Nascimento^1^, Taise Possamai-Della^1^, Thais Gois-Carvalho^1^, Jorge M. Aguiar-Geraldo^1^, Bruna Pescador^1^, João Quevedo^2^, Felipe Dal-Pizzol^3^, Samira S. Valvassori^1^

^1^Translational Psychiatry Laboratory, Graduate Program in Health Sciences, University of Southern Santa Catarina (UNESC), Criciúma, SC, Brazil.

^2^Center for Interventional Psychiatry, Faillace Department of Psychiatry and Behavioral Sciences, McGovern Medical School, The University of Texas Health Science Center at Houston (UTHealth Houston), Houston, TX, USA.

^3^Laboratory of Experimental Pathophysiology, Graduate Program in Health Sciences, University of Southern Santa Catarina (UNESC), Criciúma, SC, Brazil.

**Correspondent author:** Samira S. Valvassori. Translational Psychiatry Laboratory, Graduate Program in Health Sciences, University of Southern Santa Catarina (UNESC), Criciúma, SC, Brazil. Electronic address: [samiravalvassori@unesc.net](mailto:samiravalvassori@unesc.net).

Resource 1. Schedule of stressor agents used during the chronic treatment.

| **Day of treatment** | **Stressor used** | **Duration** | **Time of day** |
| --- | --- | --- | --- |
| Day 1 | Water deprivation | 24 h | 8:00 a.m. |
| Day 2 | Food deprivation | 24 h | 8:00 a.m. |
| Day 3 | Flashing light | 3 h | 1:00 p.m. |
| Day 4 | Restraint + cold | 2 h | 9:00 a.m. |
| Day 5 | Isolation | 24 h | 8:00 a.m. |
| Day 6 | Isolation | 24 h | 8:00 a.m. |
| Day 7 | Isolation | 24 h | 8:00 a.m. |
| Day 8 | Food deprivation | 24 h | 8:00 a.m. |
| Day 9 | Restraint | 1 h | 1:00 p.m. |
| Day 10 | Isolation | 24 h | 8:00 a.m. |
| Day 11 | Isolation | 24 h | 8:00 a.m. |
| Day 12 | Isolation | 24 h | 8:00 a.m. |
| Day 13 | No stressor applied | - | - |
| Day 14 | No stressor applied | - | - |
| Day 15 | Food deprivation | 24 h | 8:00 a.m. |
| Day 16 | Flashing light | 2.5 h | 1:00 p.m. |
| Day 17 | Water deprivation | 24 h | 8:00 a.m. |
| Day 18 | Food deprivation | 24 h | 8:00 a.m. |
| Day 19 | Flashing light | 3 h | 9:00 a.m. |
| Day 20 | Restraint + cold | 1.5 h | 1:00 p.m. |
| Day 21 | Flashing light | 3.5 h | 2:00 p.m. |
| Day 22 | Water deprivation | 24 h | 8:00 a.m. |
| Day 23 | Restraint | 2 h | 8:00 a.m. |
| Day 24 | Restraint + cold | 2 h | 1:00 p.m. |
| Day 25 | Food deprivation | 24 h | 8:00 a.m. |
| Day 26 | Flashing light | 2 h | 2:00 p.m. |
| Day 27 | Isolation | 24 h | 8:00 a.m. |
| Day 28 | Isolation | 24 h | 8:00 a.m. |
| Day 29 | No stressor applied | - | - |
| Day 30 | Food deprivation | 24 h | 8:00 a.m. |
| Day 31 | Restraint | 2 h | 3:00 p.m. |
| Day 32 | Restraint + cold | 1.5 h | 9:00 a.m. |
| Day 33 | Flashing light | 2.5 h | 1:00 p.m. |
| Day 34 | Isolation | 24 h | 8:00 a.m. |
| Day 35 | Isolation | 24 h | 8:00 a.m. |
| Day 36 | Water deprivation | 24 h | 8:00 a.m. |
| Day 37 | Food deprivation | 24 h | 8:00 a.m. |
| Day 38 | No stressor applied | - | - |
| Day 39 | Flashing light | 3 h | 8:00 a.m. |
| Day 40 | Restraint | 3 h | 2:00 p.m. |

Note: Water deprivation and food deprivation lasted 24 h starting at 8:00 a.m. Flashing light, restraint (with or without cold), and isolation were applied as indicated. This table presents the complete sequence of stressors over the experimental period.
